# Supplementary material for: Close Related Drug-Resistance Beijing Isolates of Mycobacterium tuberculosis Reveal a Different Transcriptomic Signature in a Murine Disease Progression Model
Source: Int J Mol Sci. 2022 May 5;23(9):5157. doi: 10.3390/ijms23095157 (PMC9100210; doi:10.3390/ijms23095157)
Supplement: Supplementary file 1 [file ijms-23-05157-s001.zip › Supplementary Figure S1.pdf]

Supplementary Figure S1. Venn diagram of Differentially Expressed Genes (DEG) in strain BL-323 during the infection process.

Comparison	# genes
14 vs 3	15
28 vs 3	43
28 vs 14	9

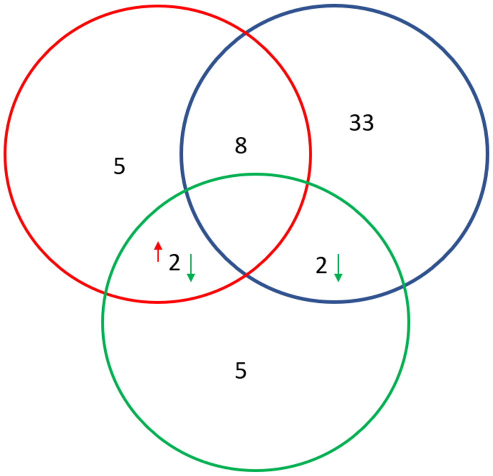

Comparison of DEG in the BL-323 strain at the day post-infection (PI). Fifteen genes were differentially expressed at D14 vs 3D PI; 43 genes were differentially expressed at D28 vs D3 PI, and 9 genes were differentially expressed at D28 vs D14 PI. Up arrows indicate over-expression, and down arrows indicate repression. Colours of circles and arrows indicate the comparison made.
