# Supplementary material for: Close Related Drug-Resistance Beijing Isolates of Mycobacterium tuberculosis Reveal a Different Transcriptomic Signature in a Murine Disease Progression Model
Source: Int J Mol Sci. 2022 May 5;23(9):5157. doi: 10.3390/ijms23095157 (PMC9100210; doi:10.3390/ijms23095157)
Supplement: Supplementary file 1 [file ijms-23-05157-s001.zip › Supplementary Figure S2.pdf]

Supplementary Figure S2. Venn diagram of DEG in strain BC-391 along days PI.

Comparison	# genes
14 vs 3	21
28 vs 3	88
28 vs 14	128
60 vs 14	1

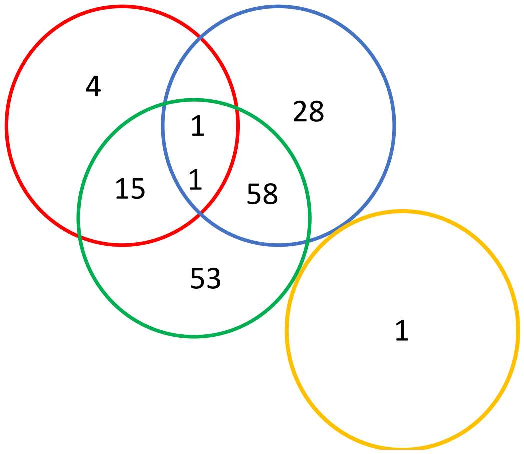

Comparison of DEG in the BL-391 strain at the day post-infection (PI). Twenty-one genes were differentially expressed at D14 vs D3 PI; 88 genes were differentially expressed at D28 vs D3 PI, 128 genes were differentially expressed at D28 vs D14 PI, and 1 gene was differentially expressed at D60 vs 14. The colours of rows in the table and circles indicate the comparison made.
