# Supplementary material for: Close Related Drug-Resistance Beijing Isolates of Mycobacterium tuberculosis Reveal a Different Transcriptomic Signature in a Murine Disease Progression Model
Source: Int J Mol Sci. 2022 May 5;23(9):5157. doi: 10.3390/ijms23095157 (PMC9100210; doi:10.3390/ijms23095157)
Supplement: Supplementary file 1 [file ijms-23-05157-s001.zip › Supplementary Figure S3.pdf]

**Supplementary Figure S3. Venn diagram of DEG in strain BC-391 vs BL-323 along days post-infection**

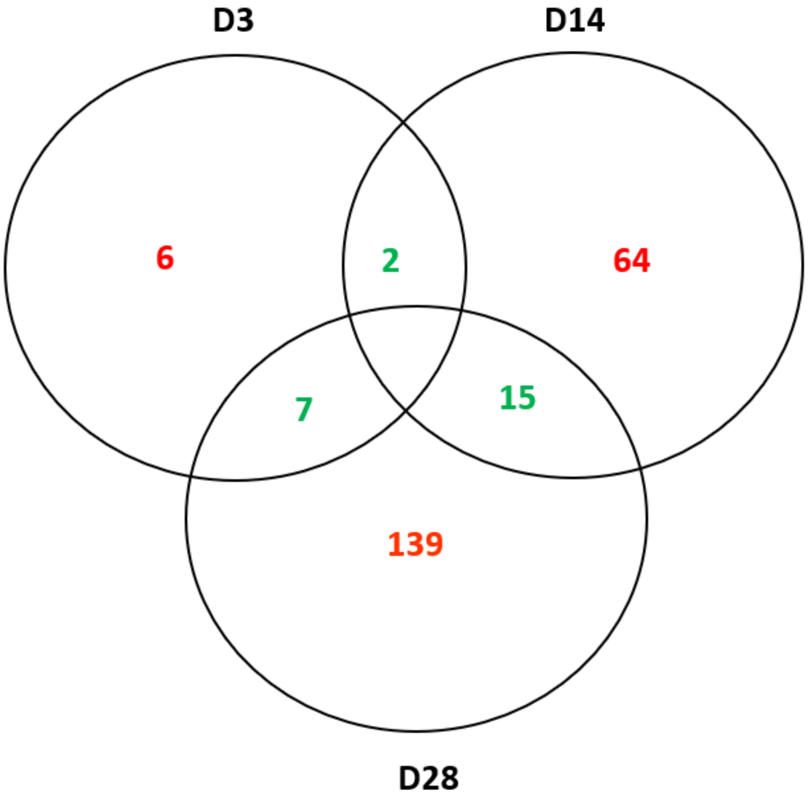


Comparison of DEG between strain BL-391 and strain BL-323 at the day post-infection (PI). Each circle represents a day PI. Red numbers indicate DEG between strains at specific day PI. Green numbers indicate DEG shares between days PI.
